# Risk factors for poor neurological recovery after anterior cervical discectomy and fusion: imaging characteristics

**DOI:** 10.1186/s13018-024-04886-7

**Published:** 2024-07-04

**Authors:** Haitao Lu, Wei Zhang, Zihao Chai, Xiubo Ge, Haiyang Yu

**Affiliations:** 1 Department of Orthopedics, Fuyang People’s Hospital of Bengbu Medical University(Fuyang People’s Hospital), 501 Sanqing Road, Fuyang, Anhui 236000 China; 2 Department of Orthopedics, Fuyang Sixth People ’s Hospital, 2019 Huaihe Road, Fuyang, Anhui 236000 China

**Keywords:** Anterior cervical discectomy and fusion, Imaging characteristics, Risk factors

## Abstract

**Background:**

Poor neurological recovery in patients after anterior cervical discectomy and fusion has been frequently reported; however, no study has analyzed the preoperative imaging characteristics of patients to investigate the factors affecting surgical prognosis. The purpose of this study was to investigate the factors that affect the preoperative imaging characteristics of patients and their influence on poor neurologic recovery after anterior cervical discectomy and fusion.

**Methods:**

We retrospectively analyzed the clinical data of 89 patients who met the criteria for anterior cervical discectomy and fusion for the treatment of single-level cervical spondylotic myelopathy and evaluated the patients’ neurological recovery based on the recovery rate of the Japanese Orthopaedic Association (JOA) scores at the time of the final follow-up visit. Patients were categorized into the “good” and “poor” groups based on the JOA recovery rates of ≥ 50% and < 50%, respectively. Clinical information (age, gender, body mass index, duration of symptoms, preoperative JOA score, and JOA score at the final follow-up) and imaging characteristics (cervical kyphosis, cervical instability, ossification of the posterior longitudinal ligament (OPLL), calcification of herniated intervertebral discs, increased signal intensity (ISI) of the spinal cord on T2-weighted imaging (T2WI), and degree of degeneration of the discs adjacent to the fused levels (cranial and caudal) were collected from the patients. Univariate and binary logistic regression analyses were performed to identify risk factors for poor neurologic recovery.

**Results:**

The mean age of the patients was 52.56 ± 11.18 years, and the mean follow-up was 26.89 ± 11.14 months. Twenty patients (22.5%) had poor neurological recovery. Univariate analysis showed that significant predictors of poor neurological recovery were age (*p* = 0.019), concomitant OPLL (*p* = 0.019), concomitant calcification of herniated intervertebral discs (*p* = 0.019), ISI of the spinal cord on T2WI (*p* <0.05), a high grade of degeneration of the discs of the cranial neighboring levels (*p* <0.05), and a high grade of discs of the caudal neighboring levels (*p* <0.05). Binary logistic regression analysis showed that ISI of the spinal cord on T2WI (*p* = 0.001 OR = 24.947) and high degree of degeneration of adjacent discs on the cranial side (*p* = 0.040 OR = 6.260) were independent risk factors for poor neurological prognosis.

**Conclusion:**

ISI of the spinal cord on T2WI and high degree of cranial adjacent disc degeneration are independent risk factors for poor neurological recovery after anterior cervical discectomy and fusion. A comprehensive analysis of the patients’ preoperative imaging characteristics can help in the development of surgical protocols and the management of patients’ surgical expectations.

## Introduction

As the global population ages, there is a rising incidence of cervical spondylotic myelopathy (CSM). The primary clinical manifestations encompass dysfunction of the upper and lower extremities, sensory abnormalities, and even urinary and fecal dysfunction. These symptoms significantly impact patients’ quality of life and frequently necessitate surgical intervention [1, 2]. Anterior cervical discectomy and fusion (ACDF) is a conventional approach for managing CSM. It involves the excision of diseased intervertebral discs and bony structures to relieve pressure on the spinal cord and/or nerve roots, as well as correct the physiological curvature of the cervical spine and restore the localized sagittal alignment [3, 4]. Despite its established role, numerous studies have documented unfavorable clinical prognosis following ACDF [5], which markedly diminishes patients’ quality of life and frequently mandates further surgical intervention. Moreover, the impact of individual factors on surgical outcomes remains a subject of ongoing debate.

On the basis of their analysis of patients’ clinical data, Li and colleagues [6] concluded that risk factors influencing postoperative clinical outcomes in patients with multilevel CSM include age, duration of symptoms, and a high cervical kyphosis angle at follow-up. Li, Wei, and their collaborators [7, 8] provided evidence of the correlation between sagittal parameters of the cervical spine (preoperative T1 tilt angle, C7 axial–sagittal distance, and postoperative C2-7 axial–sagittal distance) and postoperative outcomes. Fan [9] suggested that relying solely on patients’ preoperative symptoms and imaging parameters to predict postoperative outcomes may have limitations. Therefore, they incorporated additional factors such as patients’ smoking and alcohol history, physical examination findings, and Quality of Life Short Form 36 (QOLS) score into their analysis using cluster analysis to explore the factors affecting postoperative outcomes of CSM. They concluded that young age, low incidence of neck and shoulder pain and gait abnormalities, few positive signs of pathological reflexes, and high quality of life scores are associated with a favorable prognosis. Scerrati and colleagues [10–12] comprehensively summarized the impacts of various factors, including the utilization of cervical braces by postoperative patients and the condition of neck muscles before surgery, on the recovery of neurological function during the postoperative phase in individuals with spinal cord cervical spondylosis. The majority of the aforementioned studies focused on the preoperative symptoms and signs exhibited by patients, yet a comprehensive study of how patients’ preoperative imaging characteristics influence postoperative outcomes remains unaddressed. Commonly investigated imaging characteristics encompass cervical kyphosis [13, 14], cervical instability [15], and increased signal intensity (ISI) of the spinal cord on T2-weighted imaging (T2WI) [16]. Although many scholars have reported their correlation with postoperative outcomes, these studies overlooked the collective impact of various imaging characteristics on outcomes. Additionally, they failed to incorporate variables such as the degree of intervertebral disc degeneration, calcification of herniated intervertebral discs, and ossification of the posterior longitudinal ligament (OPLL) as imaging characteristics to be included in the study. Nevertheless, literature has documented that disc degeneration correlates with the initiation of patient symptoms [17, 18], weakening of spinal ligament integrity [19], and alterations in interlevel spinal motion [20]. Certain scholars [21] have substantiated that OPLL is an independent risk factor influencing the prognosis of respiratory function post-cervical cord injury. Furthermore, diverse types of OPLL lead to varying degrees of cervical cord impairment during cervical spine flexion and extension movements [22].

The preoperative imaging characteristics of patients significantly influence the extent of future postoperative neurological improvement and the occurrence of complications. Thus, these preoperative imaging characteristics and their effect on postoperative neurological recovery must be elucidated [23]. These imaging characteristics aid surgeons in decision-making and in managing patients’ expectations regarding surgical outcomes. However, clinical studies that comprehensively analyze the impact of patient imaging characteristics on postoperative outcomes are lacking. Thus, the objective of this study was to analyze the influence of patients’ preoperative imaging characteristics on adverse neurological outcomes following ACDF by employing univariate and binary logistic regression analyses.

## Patients and methods

### Study populations

Approval for this study was obtained from our institutional review board. Data retrieval involved gathering information from the medical records and imaging data of patients who underwent anterior cervical discectomy and fusion procedures performed by the same spinal surgeon for the treatment of single-level CSM at our institution. The inclusion criteria were as follows: (1) clear diagnosis of CSM based on the patient’s medical history, symptoms, signs, and imaging examinations, with evidence of progressive worsening of spinal cord compression symptoms; (2) confirmation of spinal cord compression alignment with investigative symptoms, and identification of single-level spinal cord compression on CT and MRI scans; and (3) availability of complete clinical and imaging data.

Exclusion criteria were as follows: (1) medical history of cervical spine surgery; (2) patients with cervical spinal cord injury, tumor history, neurological lesions, ankylosing spondylitis, rheumatoid arthritis, diffuse idiopathic cranial hypertrophy, or other conditions; and (3) inadequate acquisition of imaging data and functional scoring data, or indistinct depiction of significant bone markers on radiological imaging.

We comprehensively examined the clinical and imaging-related profiles of all patients and assessed potential factors influencing postoperative neurological outcomes. Moreover, each patient underwent follow-up exceeding 12 months, during which neurological function was assessed using the Japanese Orthopaedic Association (JOA) score at preoperative and final follow-ups. The JOA recovery rate served as a metric for assessing the restoration of postoperative neurological function. The recovery rate was determined using the Hirabayashi method as follows: recovery rate % = (last follow-up score - preoperative score) / (17 [highest JOA score] - preoperative score), expressed as a percentage. Previous research indicates that the optimal recovery time frame typically falls within 12 months post-surgery [24], with neurological recovery deemed satisfactory if the JOA recovery rate is ≥ 50% [25].

### Clinical and radiologic assessments

Patient data collection included age, sex, body mass index (BMI), symptom duration, preoperative and final follow-up JOA scores, and assessment of imaging characteristics: presence of cervical kyphosis (yes or no), cervical instability (yes or no), OPLL (yes or no), calcification of herniated intervertebral discs (yes or no), ISI of the spinal cord on T2WI (yes or no), and degree of adjacent level disc degeneration (cranial and caudal to the fused level).

**Fig. 1:**
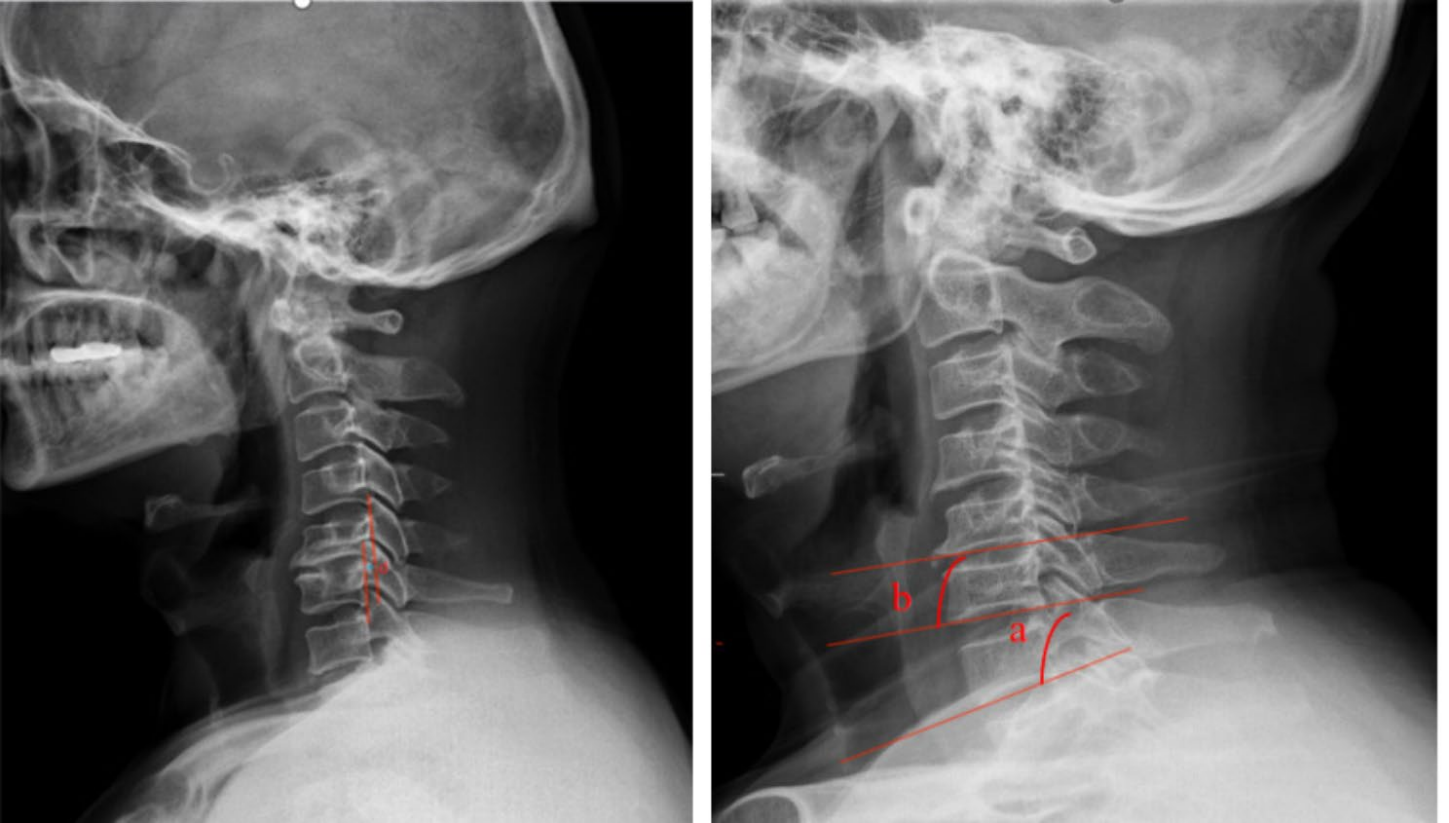
Translational instability is defined as more than 3.5 mm horizontal displacement of one vertebra in relation to an adjacent vertebra measured on lateral roentgenograms (d ≥ 3.5 mm, left). Rotational instability is defined as more than 11 degrees rotational difference from that of either adjacent vertebra (a–b ≥ 11°, right)

**Fig. 2:**
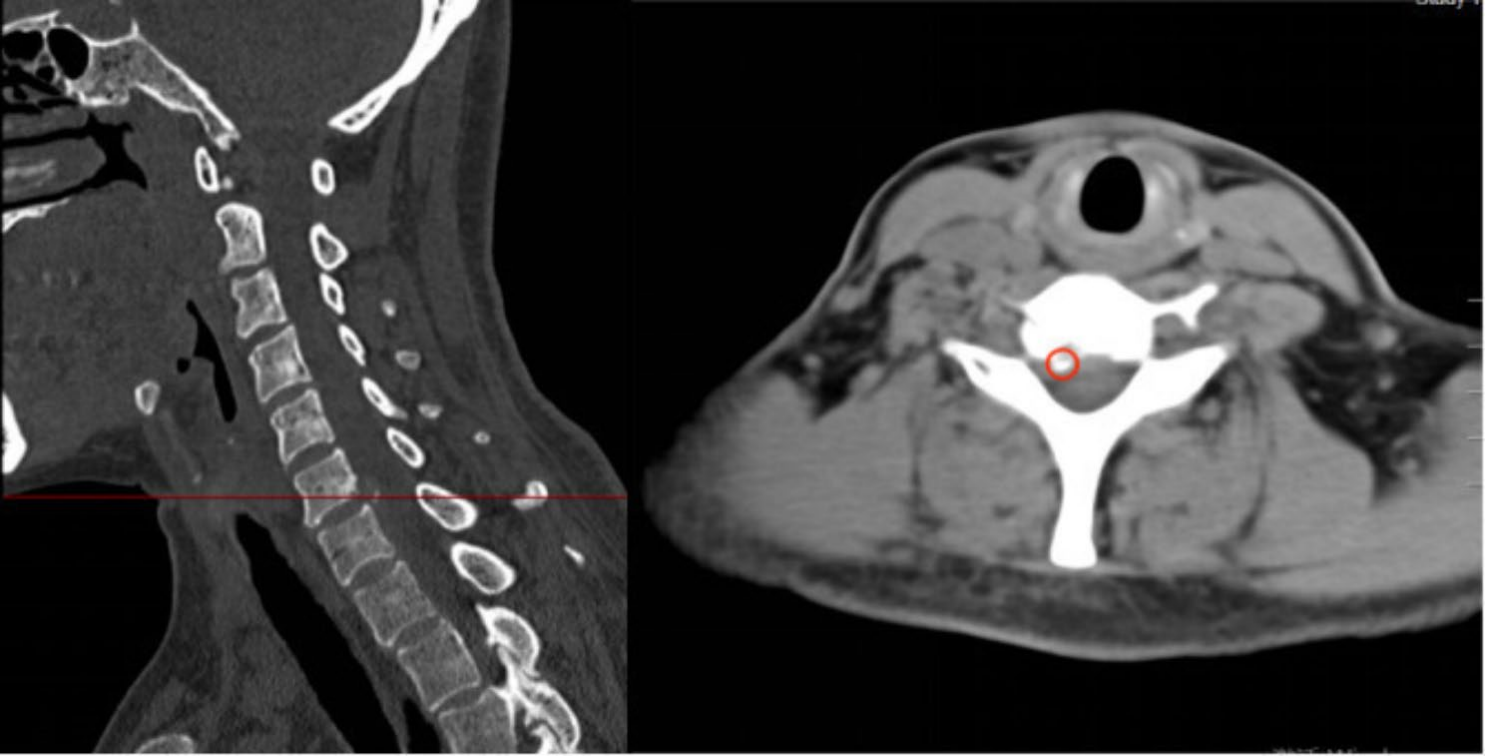
Portion circled in red is calcification of the herniated portion of the disc

**Fig. 3:**
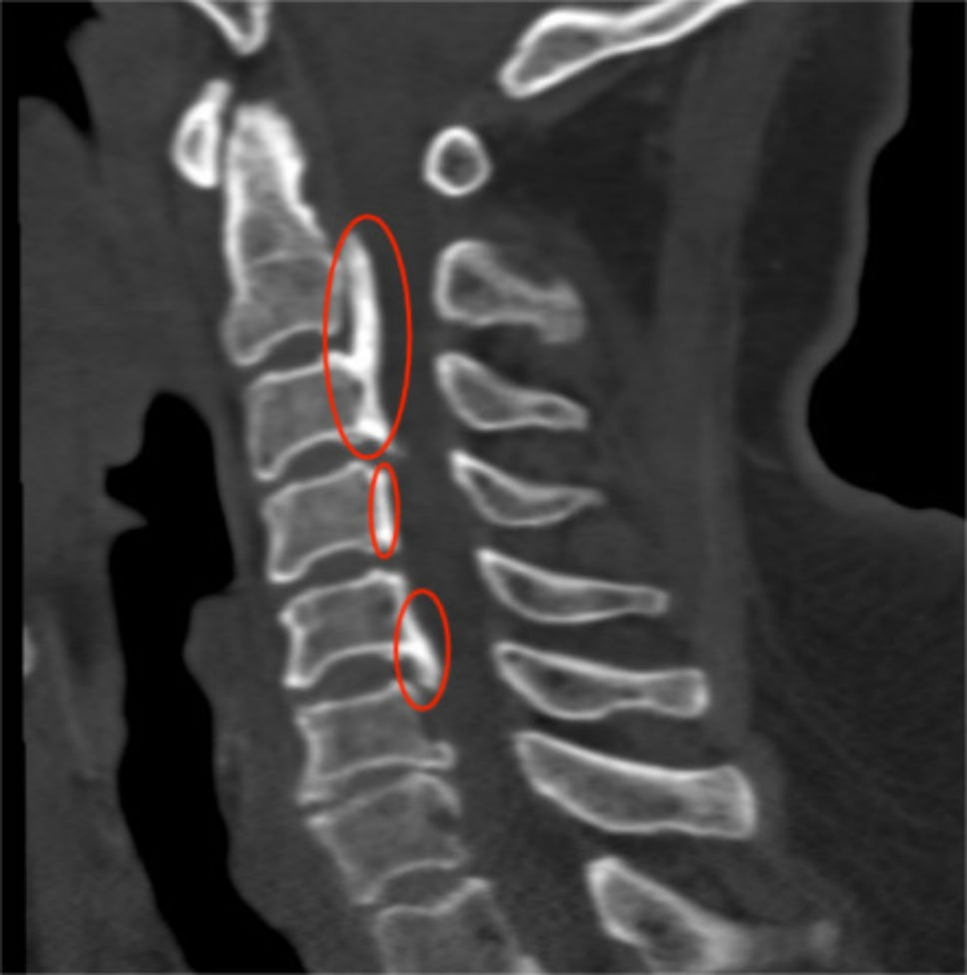
Portion circled in red is the posterior longitudinal ligament, which has undergone ossification

**Fig. 4:**
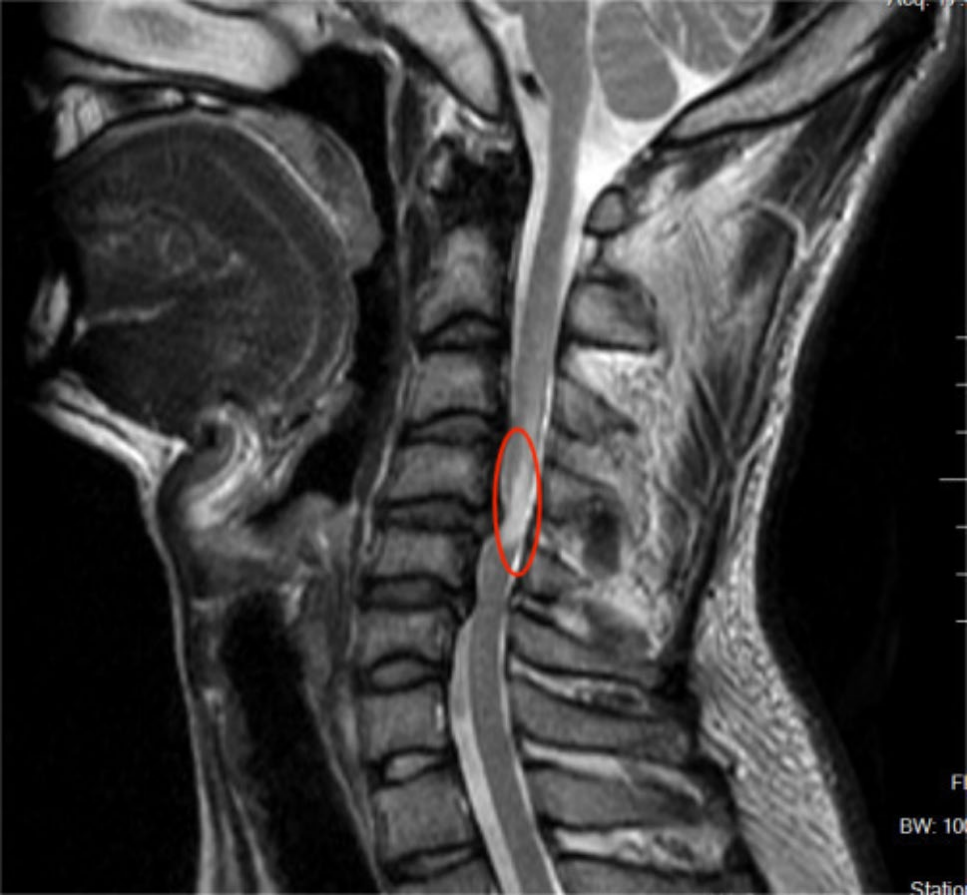
Portion circled in red is the spinal cord for increasing signal

Prior to surgery, all patients underwent preoperative lateral cervical radiographs for the assessment of cervical instability and kyphosis; cervical spine CT scans for detecting OPLL and calcification of herniated intervertebral discs; and cervical spine MRI for evaluating ISI of the spinal cord on T2WI and adjacent level disc degeneration. ①Symptom duration [26]: Grade I: symptom duration within 3 months; Grade II: symptom duration between 3 months and 1 year; Grade III: symptom duration exceeding 1 year. ②Cervical instability: (1) Translational instability: horizontal displacement of 1 vertebra exceeding 3.5 mm relative to adjacent vertebrae, measured on lateral radiographs; (2) Rotational instability: rotation difference exceeding 11° of any adjacent vertebrae, measured on lateral radiographs [27](Fig. 1).③Cervical kyphosis: C2–C7 angles exceeding 5°(Jackson physiologic stress line method): the angle formed between the parallel lines of C2 and the posterior margin of C7 vertebrae. ④Calcification of herniated intervertebral discs: calcification of herniated cervical disc tissue (Fig. 2). ⑤Ossification of the posterior longitudinal ligament: ossification of the posterior longitudinal ligament on the posterior aspect of the cervical vertebral body (Fig. 3). ⑥ISI of the spinal cord on T2WI: According to Yukawa’s grading criteria [28],spinal cord signal intensity increase was categorized into three groups based on sagittal T2-weighted images as follows: Grade 0, absence; Grade 1, light (obscure); and Grade 2, intense (bright). Grade 0 was defined as no increased signal intensity of the spinal cord on T2WI, and Grades 1 and 2 indicated increased signal intensity of the spinal cord on T2WI (Fig. 4). ⑦Degree of disc degeneration in the adjacent levels [29]: assessment of disc grading in the neighboring levels adjacent to the fused levels according to the Pfirrmann grading scale: grades I–V.

### Surgical procedure

The patient was placed in a supine position under general anesthesia, and the surgical area was disinfected and draped. A transverse or oblique incision was made on the right side of the neck to expose the responsible level. The anterior longitudinal ligament was longitudinally incised; after the retractor was placed to expand the space, the intervertebral disc tissue was removed, and the upper and lower cartilaginous endplates and osteophytes were scraped off from the intervertebral space. The longitudinal ligament was resected after nibbling and biting, and decompression was performed by traversing in all directions; the dura mater bulging was explored and found to be uncompressed. A suitable cage size was selected according to the height of the adjacent intervertebral space. After filling the cage with osteophytes and crushed bones, it was implanted into the intervertebral space. A titanium plate and screws of appropriate lengths were selected for fixation, and the positions of the cage and titanium plate were confirmed to be satisfactory using a C-arm X-ray machine for fluoroscopy. The incision was irrigated, a drainage tube was placed, the incision was closed layer by layer, and sterile dressings were applied.

### Statistical analysis

SPSS version 26 (IBM SPSS Statistics 26.0, IBM Corporation, Armonk, NY, USA) was used to perform all statistical analyses. The normality of the measurement data was tested using the Shapiro–Wilk method (*P* > 0.05). All data conformed to a normal distribution, so the measurement data are described by the mean ± standard deviation (mean ± SD). Comparisons between the two groups were analyzed using independent samples t-test. The Chi-square test was used to compare groups regarding categorical variables. Multivariate correlation analysis was performed using all variables identified as significant at the *p* ≤ 0.05 level on univariable analysis to identify the risk factors associated with poor neurological outcomes.

## Result

### Univariate analysis results

A total of 89 patients (mean age 52.56 ± 11.18 years; comprising 44 males and 45 females) were enrolled in this study, with a mean BMI of 24.89 ± 3.06 kg/m^2^. The average preoperative and final follow-up JOA scores were 11.39 ± 1.23 and 15.02 ± 1.14, respectively, resulting in a mean JOA recovery rate of 65.26%. On the basis of the JOA recovery rate, patients were stratified into two groups: 69 patients (77.5%) were classified in the good group, whereas 20 patients (22.5%) were categorized in the poor group.

### JOA recovery rate ≥ 50% versus JOA recovery rate < 50%

Clinical parameters included mean age (51.07 ± 9.565 years vs. 57.70 ± 14.63 years, *P* = 0.019), gender ratio F/M (33:36 vs. 12:8, *P* = 0.338), BMI (24.83 ± 2.88 kg/m^2^ vs. 25.11 ± 3.68 kg/m^2^, *P* = 0.728), duration of symptoms (I: II: III 34:14:21 vs. 6:3:11, *P* = 0.128), and preoperative JOA score (11.45 ± 1.059 vs. 11.75 ± 1.734, *P* = 0.497). Imaging characteristics included cervical kyphosis (yes/no 28:41 vs. 11:9, *P* = 0.252), cervical instability (yes/no 23:46 vs. 10:10, *P* = 0.174); OPLL (yes/no 7:62 vs. 7:13, *P* = 0.019), calcification of herniated intervertebral discs (yes/no 7:62 vs. 7:13, *P* = 0.019), ISI of the spinal cord on T2WI (yes/no 20:49 vs. 18:2, *P* = 0.000), degree of disc degeneration in cranial adjacent levels (III: IV: V 20:40:9 vs. 0:9:11, *P* = 0.000), and degree of disc degeneration in caudal adjacent levels (III: IV: V 31:31:7 vs. 2:18:8, *P* = 0.001).

### Logistic regression analysis of good and poor groups

Table 1 illustrates significant differences in age, OPLL, calcification of herniated intervertebral discs, ISI of the spinal cord on T2WI, and degree of disc degeneration in the adjacent levels on the cranial and caudal side between the good and poor groups (*P* < 0.05). By contrast, no statistically significant differences were observed between the two groups in terms of gender, BMI, symptom duration, cervical kyphosis, and cervical instability (*P* > 0.05). Univariate analysis revealed that six indicators were identified as independent variables, namely, age, OPLL, calcification of herniated intervertebral discs, ISI of the spinal cord on T2WI, and degree of disc degeneration in the adjacent levels (cranial and caudal) adjacent to the fused level. Subsequently, binary logistic regression analysis was conducted, utilizing the JOA recovery rate as the dependent variable (Table 2), with the model being assessed by the Hosmer–Lemeshow test (*P* = 0.798). Logistic regression analysis revealed a significant association among ISI of the spinal cord on T2WI, a high degree of disc degeneration in the cranial adjacent levels, and the JOA recovery rate. In particular, ISI of the spinal cord on T2WI and a high degree of disc degeneration in the cranial adjacent level were identified as independent risk factors affecting the JOA recovery rate (OR > 1). Specifically, patients with ISI of the spinal cord on T2WI were 24.947 times more likely to experience poor neurological recovery compared with those without this signal (OR = 24.947). Similarly, patients with a high grade of disc degeneration in the cranial adjacent level were 6.260 times more likely to encounter poor neurological recovery relative to those with a lower grade of disc degeneration in the same level (OR = 6.260).

**Table 1:** Comparison of factors in the good and poor groups

Variables	JOA recovery Rate ≥ 50% (*n* = 69)	JOA recovery Rate<50 (*n* = 20)	X^2^/t	*P*
Age (years) Female/male BMI (kg/m^2^) Preoperative JOA score duration of symptoms (I:II:III) Cervical kyphosis (Y/N) Cervical instability (Y/N) OPLL (Y/N) Pfirrmann classification Cranial side (III:IV:V) Caudal side (III:IV:V) ISI on T2WI (Y/N) Calcification discs (Y/N) Final follow-up time (month)	51.07 ± 9.565 33/36 24.83 ± 2.88 11.46 ± 1.06 34:14:21 28:41 23:46 7:62 20:40:9 31:31:7 20:49 7:62 27.13 ± 11.372	57.70 ± 14.63 12/8 25.11 ± 3.68 11.17 ± 1.73 6:3:11 11:9 10:10 7:13 0:9:11 2:18:8 18:2 7:13 26.05 ± 10.546	2.395 0.919 0.348 -0.690 4.111 1.310 1.846 5.473 17.728 13.388 23.593 5.473 -0.380	0.019* 0.338 0.728 0.497 0.128 0.252 0.174 0.019* 0.000* 0.001* 0.000* 0.019* 0.705

*There was significant differences between the two groups (*P* < 0.05)

Y, yes; N, no;

**Table 2:** Logistic regression analysis of good and poor groups

Variables	β	OR	95% CI	*P*
Age (years) OPLL (Y/N) Pfirrmann classification Cranial side (III:IV:V) Caudal side (III:IV:V)	0.025 1.747 1.834 0.290	1.025 5.738 6.260 1.337	0.966–1.088 0.917–35.899 1.091–35.924 0.363–4.930	0.415 0.062 0.040* 0.663
ISI on T2WI (Y/N) Calcification discs (Y/N)	3.217 0.937	24.947 2.553	3.864-161.048 0.382–17.072	0.001* 0.334

*There was significant differences between two groups (*P* < 0.05)

Y, yes; N, no;

**β**, Coefficient estimation; OR, odds ratio; CI, confidence interval

## Discussion

The imaging characteristics and clinical data of the patients were comprehensively analyzed via logistic regression to control for the influence of cervical instability and symptom duration on the poor prognosis of patients. Our study demonstrated that ISI of the spinal cord on T2WI and high degree of cranial adjacent disc degeneration were independent risk factors for poor neurological recovery after anterior cervical discectomy and fusion.

Nevertheless, the influence of preoperative ISI of the spinal cord on postoperative outcomes remains under debate. Some researchers [30] quantitatively analyzed the preoperative T2WI spinal cord signals of 112 patients with ACDF patients by signal intensity ratio method, and they found that the ISI of the spinal cord on preoperative T2WI is an important factor to predict postoperative efficacy, which was consistent with our results. Yagi [31] investigated preoperative MRI data from 71 patients subjected to cervical laminoplasty. Their analysis revealed that patients exhibiting ISI of the spinal cord on T2WI manifested markedly diminished rates of JOA improvement postoperatively. Furthermore, they experienced substantially poor long-term follow-up efficacy as the area of high signal intensity expanded postoperatively. Yu et al. [32]. conducted a comparative study on preoperative ISI of spinal cord among patients undergoing cervical laminoplasty. Their findings indicated no discernible disparity in the rate of postoperative JOA improvement between patients with and without ISI spinal cord signal. Matsumoto et al. [33]. investigated the prognosis of patients with conservatively managed cervical compressive myelopathy. Their investigation revealed that ISI of the spinal cord on preoperative T2WI is not correlated with unfavorable efficacy of conservative therapy. Alafifi et al. [34]. concluded that patients who have a high intramedullary signal change on T2WI but do not exhibit clonus or spasticity may have a positive surgical outcome and potential reversal of the MRI abnormality. Additionally, Lu [15] found no significant correlation between ISI of the spinal cord on T2WI and poor prognosis.

The findings from prior investigations regarding the correlation between ISI of the spinal cord on T2WI and surgical prognosis in patents with CSM differed because of differences in study design, control of confounding variables, and criteria for evaluating surgical efficacy. To mitigate the intricacy associated with grading changes in spinal cord signal and mitigate potential confusion and misinterpretation, this study employed the Yukawa grading criteria, which are extensively applied in clinical settings. Moreover, we streamlined the criteria into binary categorization (yes or no). Remarkably, 90% of patients in the poor group exhibited ISI of the spinal cord on T2WI in our study. We believe that the ISI of the spinal cord on T2WI mirrors the underlying pathology of spinal cord injury, including edema, gliosis, demyelination, and spinal cord chondromalacia [16]. These changes may indicate irreversible damage. ISI of the spinal cord on T2WI might also indicate secondary spinal cord edema due to acute spinal cord ischemia, leading to cervical cord glucose dysfunction and insufficient blood supply [16], which can affect the neurological outcome of patients after cervical surgery. Yukawa et al. [28]. also believe that ISI of the spinal cord on preoperative T2WI signifies a manifestation of severe, chronic spinal cord injury, reflecting the small potential of neurological recovery in postoperative patients.

Our results not only demonstrated that ISI of the spinal cord on T2WI is an independent risk factor for patient outcome but also revealed that a high degree of cranial adjacent disc degeneration can adversely affect postoperative efficacy. Degeneration of the intervertebral discs results in diminished stability and alterations in biological stress within the cervical spine, representing an initial indication of degenerative transformations in the spinal column [35]. As cervical spine fusion surgery continues to advance, there is a growing emphasis on investigating disc degeneration in levels adjacent to fused levels. Several studies have indicated [36] that the degeneration of the disc in the adjacent level is an independent risk factor for adjacent segment disease after ACDF.

We posit potential reasons for poor postoperative efficacy resulting from significant disc degeneration, which are as follows: (1) Abnormal muscle activities and bodily posture may ensue under disc herniation or severe degenerative processes. Upon such occurrences, the body gradually hardens the muscles in the degenerated areas and decreases the range of motion, whereas healthy segments compensate to maintain a similar range of motion in the spine [37]. After surgery, fusion of the responsibility level and reduction in intervertebral space height resulting from adjacent level disc degeneration require compensatory mechanisms to preserve mobility in the cervical spine and maintain head–neck equilibrium. Abnormal muscle activities and bodily posture may be employed for compensation, thereby contributing to poor postoperative efficacy. (2) Aseptic inflammation may occur, given that recent investigations have elucidated [38] that inflammatory cytokines are overexpressed within the cervical disc. This inflammatory state elicits a variety of pathogenic responses, including cellular senescence, apoptosis, inward proliferation of nerves and blood vessels, and discogenic pain, potentially impeding the recovery of neurological function in patients. (3) Change in stress distribution may occur, as preoperative degeneration of adjacent discs is correlated with heightened postoperative pressure in the neighboring discs [39]. Subsequently, intervertebral discs in adjacent levels postoperatively transmit intramedullary pressure. The adjacent level intervertebral disc transfers pressure from the nucleus pulposus to the annulus fibrosus, which exacerbates annulus fibrosus degradation. Biomechanical alterations induce cytological changes within intervertebral discs. The emergence of such abnormal stress patterns may impede neurological function recovery and cause adjacent segment disease. (4) Release of inflammatory mediators due to preoperative cervical disc degeneration contributes to the thickening of subcutaneous adipose tissues around the neck. This phenomenon prompts the secretion of cytokines, including IL-1 and TNF, from adipose tissue. Such cascading events may induce secondary adverse effects, including impaired cellular diffusion within the intervertebral disc, reduced intervertebral disc hydration, and diminished weight-bearing capacity of the intervertebral disc. Consequently, these processes can impede the postoperative recovery of neurological function in patients [40]. Nevertheless, some scholars also hold the opposite view; their research [41] showed that the degree of disc degeneration does not necessarily correlate with poor postoperative efficacy in patients. Diminished cervical mobility resulting from disc degeneration similar to the fusion effect of surgery may have a positive impact on surgical outcome. At present, few studies have examined the correlation between disc degeneration and postoperative outcomes, warranting further investigation into the precise underlying mechanisms.

This study had some limitations, as follows: (1) subjectivity and qualitative assessment inherent in the Pfirrmann grading system may introduce observer bias; (2) limited imaging indicators necessitate a large sample size and imaging parameters in the regression model to enhance model fitting; and (3) all cases were from the same hospital, so there are some restrictions on the extrapolation of conclusions. In the future, the source of cases can be expanded for multi-center study.

## Conclusions

ISI of the spinal cord on T2WI and high degree of cranial adjacent disc degeneration are independent risk factors for poor neurological recovery after anterior cervical discectomy and fusion. Comprehensive analysis of the patients’ preoperative imaging characteristics can help surgeons develop a surgical plan and manage the patients’ surgical expectations.

## Data Availability

No datasets were generated or analysed during the current study.
